# A Systematic Review of the Causes, Consequences, and Solutions of Emergency Department Overcrowding in Saudi Arabia

**DOI:** 10.7759/cureus.50669

**Published:** 2023-12-17

**Authors:** Afnan Almass, Meshari M Aldawood, Hessah M Aldawd, Saad I AlGhuraybi, Abdulrahman A Al Madhi, Mai Alassaf, Alwaleed Alnafia, Abdulrahman I Alhamar, Abdulaziz Almutairi, Feras Alsulami

**Affiliations:** 1 Emergency Medicine, Ministry of Health, Riyadh, SAU; 2 Emergency Medicine, Imam Mohammad Ibn Saud Islamic University, Riyadh, SAU; 3 Emergency Medicine, Al Iman General Hospital, Riyadh, SAU; 4 Medicine and Surgery, Alfaisal University College of Medicine, Riyadh, SAU; 5 Medicine, Imam Mohammad Ibn Saud Islamic University, Riyadh, SAU; 6 Medicine and Surgery, AlMareefa University, Riyadh, SAU; 7 General Practice, Alahsa Health Cluster, Alahsa, SAU; 8 Emergency Medicine, King Khalid Military City Hospital, Hafar Albatin, SAU

**Keywords:** saudi arabia, prolonged care, telemedicine, emergency department, emergency crowding

## Abstract

This study aims to investigate and address the issue of emergency department (ED) overcrowding, a significant problem worldwide. The study seeks to understand the impacts of ED overcrowding on emergency medical healthcare services and patient outcomes. This systematic review follows the PRISMA flow diagram and the guidelines of the Cochrane Handbook. We systematically reviewed the causes and solutions of emergency department overcrowding. We went through Google Scholar, the National Center for Biotechnology Information, the British Medical Journal, Science Direct, Ovid, Cochrane, the Saudi Journal of Emergency Medicine, Medline, and PubMed as databases. Our criteria were articles done in Saudi Arabia from 2012 to 2022. One hundred and ninety-six (196) research papers were extracted; only 28 articles met our paper inclusion-exclusion criteria. The result of these papers regarding causes, consequences, and solutions was that non-urgent and returned visits lacked knowledge of PHC, triad, and telemedicine services. Prolonged LOS is due to slow bed turnover, laboratory and consultation time, and physical response to the final decision resulting in burnout staff, wrong diagnoses, and management plans. The crowding issues can be resolved by awareness, PHC access, triad systems, and technological and telemedicine services. High demand for emergency treatment should not be a hindrance to quality treatment. Physical, technological, and strategic measures should be put in place to fight the crowding problem in EDs in Saudi Arabia, as it may cause adverse effects such as transmission of diseases and death of patients.

## Introduction and background

Overcrowding in emergency departments (EDs) has been a topic of study across the globe, and it has been called the single greatest threat to the security of the healthcare system everywhere [1]. A National United States survey in 2001 reported crowding in EDs as high as 91% of total patients who visit the hospital [2]. A national cross-sectional study in Sweden found that 12 out of 37 hospitals had high occupancy rates [3]. EDs are a safety net and are open 24 hours a day, making them a fundamental part of public health, and increased crowding can be a detriment to that status [4]. ED crowding affects patients by delaying their diagnosis and treatment, resulting in decreased quality of care, causing poor outcomes and increased mortality [5]. There is also a modest increase in the length of stay and costs for admitted patients [4,6-8]. ED crowding is associated with poor quality of care in patients with severe pain [2]. The level of crowding is affected by the time of the day and the day of the week. Overcrowding adversely impacts patient care and increases the number of patients who leave without being seen (LWBS) [9].

ED crowding affects individual patients, healthcare systems, and communities; evidence shows increased emergency healthcare service utilization because of the increased rates of accidental injuries. However, the capacity of the emergency healthcare systems has not been well developed to respond to such high demand, because creating a balance between emergency services and the required resources is challenging [10]. Hospitals need to address interventions and policies to address this significant problem [11]. ED crowding was not independently associated with mortality (odds ratio (OR) 0.94, 95% confidence interval), but tended to be associated with a higher incidence of hospital-acquired pneumonia [12].

The Society for Academic Emergency Medicine has concluded that a standardized approach using more simple methods of both time intervals (flow) and patient counts (nonflow) would be most beneficial. They also suggested that the usefulness of the measures would be determined by the extent to which they inform priority outcomes, including clinical outcomes and patient safety [13]. The introduction of geriatric emergency medicine and the expansion of emergency medicine training may be associated with undesirable consequences contributing to ED crowding. Enhancing primary care effectively reduced ED crowding [14]. Increased mortality in the hospital, as well as moderate increases in patients' duration of stay and overall healthcare expenses, were linked to periods of peak ED congestion [8].

At the same time, reported solutions focused on efficient patient flow within the ED. Individual hospitals must have full-capacity protocols with agreed and defined triggers. These protocols recruit support from inpatient services, focus the minds of bed and case managers, and set clearly defined thresholds and actions; various interventions reduce crowding in ED. Ensuring patients are seen early by a senior emergency physician who can "front-load" is helpful. Training nursing staff to order X-rays at triage is helpful and cuts the patient's stay by around 20 minutes [2,6]. The study aims to investigate and address ED overcrowding, a significant problem worldwide.

## Review

Methods

This systematic review follows the PRISMA flow diagram and the guidelines of the Cochrane Handbook.

Inclusion criteria

(1) All primary study designs that evaluate the overcrowding in EDs in Saudi Arabia.

(2) Articles published in the last 10 years (2012-2022).

(3) Peer-reviewed articles originally published in English.

Exclusion criteria

(1) Any research that is not related to Saudi Arabia.

(2) Studies published before 2013.

(3) Articles not relevant to our objectives.

Search strategy

We systematically reviewed the causes and solutions of ED crowding in Saudi Arabia. Between August 17 and September 24, 2022, we searched Google Scholar, National Center for Biotechnology Information, British Medical Journal, Science Direct, Ovid, Cochrane, Saudi Journal of Emergency Medicine Medline, and PubMed databases. We gathered articles done in Saudi Arabia in the last 10 years.

Search, keyword searched, and study selection

The included articles were reviewed in three stages. The initial stage involved utilizing EndNote Software to import the findings from electronic databases onto a Microsoft Excel sheet. The second step involved two independent authors assessing the article titles and abstracts entered into the Excel sheet. The included citations from Step 2 were subjected to full-text screening in the third stage. In addition, we manually checked the included publications' references for any potential overlooked investigations.

Selection of the studies and data extraction

All reviewers collected the articles to create a literature review and then worked together to include articles that addressed ED crowding, including causes, effects, solutions, or all three in Saudi Arabia. Any article that did not address these variables or was done outside Saudi Arabia was excluded from the final review. Articles were also excluded if they were published in more than 10 years or when they were not related documents and unavailable as full text. Duplicates were also removed manually at this stage. From each article in the final list, we extracted data about causes, effects, and solutions to ED crowding; then, we manually grouped information on each point to create unified data.

Results

Results of the Literature Search

**Figure 1:**
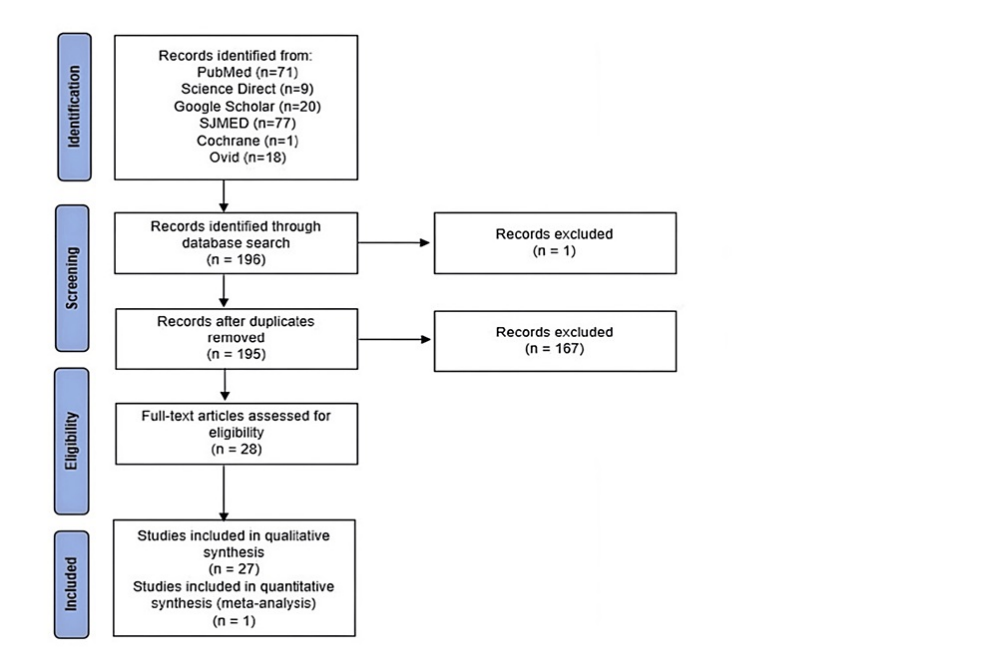
PRISMA 2009 flow diagram

The search process followed the PRISMA guidelines, shown below in the PRISMA flow diagram (Figure 1).

Out of 196 articles, 138 were irrelevant, 18 were not published in Saudi Arabia, seven did not meet our criteria, four were published in more than 10 years, and one duplicated article, and 28 were included and met our paper inclusion-exclusion criteria.

Characteristics of the Included Studies

The result of these papers regarding causes, consequences, and solutions was that non-urgent and returned visits lacked knowledge of PHC, triad, and telemedicine services. Prolonged LOS is due to slow bed turnover, laboratory and consultation time, and physical response to the final decision, and leads to burnout staff, wrong diagnoses, and management plans. Crowding issues are resolved by awareness, PHC access, triad systems, and technological and telemedicine services.

Qualitative synthesis

Nasradeen et al. [15] found a high incidental rate of unscheduled return visits to the ED within 72 hours. To address this, the admission and discharge policies should be reassessed with more attention and preventive treatment to reduce the recurrence of common complaints. Non-urgent visits to EDs are common, leading to a significant burden on the ED [16]. The study suggests that CTAS V cases can be safely triaged away to PHC and CTAS IV cases can be either triaged to PHC or the urgent care center to decrease the ED burden. Astek et al. [17] found that a large proportion (81%) of ED visits were non-urgent. Still, there is no association between non-urgent visits and patients' mortalities, suggesting that non-urgent cases may not directly impact patients' outcomes in terms of mortality. Alrabiah et al.'s [18] retrospective validation study showed that the Sydney Triage Admission Risk Tool (START) model is highly effective in predicting patient disposition in the ED at KKUH in Riyadh, Saudi Arabia. A cross-sectional study [19] revealed that many individuals lack an understanding of the triage system and waiting time in the ED. Participants showed an increased anticipation of negative test outcomes, indicating lower satisfaction with the ED experience. However, they demonstrated a decent understanding of the factors influencing priority in the triage system. Evidence-based strategies, including structural changes, technological enhancements, and procedural improvements, were identified to reduce ED crowding at King Faisal Specialist Hospital and Research Center [20]. Factors influencing crowding and performance in the ED were identified, leading to the development of indicators for assessment and improvement [21].

Point of care testing (POCT) at triage in a crowded ED showed potential benefits, as it led to immediate patient transfer, changed triage acuity and was found helpful by triage nurses in most cases, enhancing patient safety [22]. A significant association was found between ED crowding status and patients' length of stay in a university hospital, highlighting the impact of crowding on patient outcomes [23]. Al-Wathinani et al. [24] showed that patient satisfaction in the ED is linked to employee performance and ED flow. Overcrowding negatively impacts employee performance and leads to patient dissatisfaction with healthcare outcomes. The mHealth application "EDWat" was developed to improve ED waiting times and care quality. Patients can use the application to find nearby EDs with shorter wait times and higher quality ratings, potentially reducing wait times and enhancing care quality in EDs [25]. Alfaleh et al. [26] focused on the role of telemedicine services, including the Sehha application and 937 medical call centers, in changing users' intentions for visiting EDs in Saudi Arabia. The findings can provide insights into the potential impact of telemedicine on ED utilization.

Al-Onazi et al. [27] aimed to decrease loss in an acute care unit at King Abdullah Specialized Children's Hospital. By implementing the pediatric rapid assessment and management (PRAM) methodology, improvements were observed in reducing wait times and length of stay for patients triaged as Level III (potential urgency or severe pain) and Level IV (moderate pain). The average evaluation time in the I.A.R. and the median time spent in a PRAM bed were significantly reduced, contributing to more efficient patient care in the ED. Khalifa et al. [28] identified deficiencies and suggested potential ER performance improvements at King Faisal Specialist Hospital and Research Center. Solutions such as team triaging, patients' palmar scanning, and placing a physician in triage may enhance emergency room efficiency. Abudan et al. [29] focused on crowding measurement tools in pediatric emergency rooms. Four research studies on pediatric ED crowding metrics were analyzed, indicating promising findings. However, the subject is still in its early stages, and further research is needed to compare crowding assessments across different pediatric EDs. A retrospective, observational study by Abudan et al. [29] evaluated the effect of nursing patient flow coordinators on the LOS of boarded patients in the ED. The intervention did not reduce LOS for patients hospitalized in a ward, possibly due to worsening signs of hospital congestion. To achieve greater effects on LOS, the intervention should have been combined with ED input and output procedures enhancements. Bukhari et al. [30] provided insights into factors associated with prolonged LOS, such as laboratory and consultation time, patient admission, and physical response to the final decision. This data can guide future projects to shorten LOS and improve patient satisfaction. Alfaleh et al. [26] estimated the number of patients intending to visit the emergency room using the Sehha smartphone application and 937 medical call centers in Saudi Arabia. The telemedicine services effectively reduced the number of patients visiting the ED by providing medical advice for minor medical issues, showcasing the potential of such services in managing ED utilization.

Alsalamah et al. [31] investigated predictors of increased LOS in patients admitted to the ED of King Abdullah bin Abdulaziz University Hospital in Riyadh. LOS was higher in patients over 40 years old and in females compared to males. Further research is needed to explore the underlying causes of gender differences related to extended LOS in Saudi Arabia. Bakarman et al. [32] evaluated all patient visits to the ED of King Fahd General Hospital in Jeddah. The findings revealed that a significant portion of ED visits were non-emergency cases, and the top three reasons for visits were trauma or RTA, fever, and GIT problems. The study suggested implementing the "fast track" concept to promptly treat patients with less severe conditions. Al-Otmy et al. [33] explored factors associated with non-urgent visits to the ED in a tertiary hospital in Western Saudi Arabia. A significant proportion of visits were classified as non-urgent, with reasons including the belief that the illness was urgent, convenience, and lack of available treatments at PHC facilities. The study recommended promoting primary care services for less urgent medical issues. Khubrani et al. [34] investigated potential relationships between ED overcrowding and increased patient mortality rates at a teaching hospital in the Eastern Province of Saudi Arabia. The study identified age groups with higher fatality rates during overcrowding periods. Implementing the study's suggested measures to raise public awareness and expand ED bed capacity may improve patient outcomes.

Astek et al. [17] evaluated the relationship between the ratio of non-urgent visits and mortality at King Khalid National Guard Hospital in Jeddah. A large proportion of ED visits were non-urgent, but there was no association between non-urgent visits and mortality. Ahmed et al. [35] explored the causes of non-urgent ED visits at King Fahad Hospital-Albaha, Saudi Arabia. A significant number of patients were categorized as non-urgent, and most of them had no urgent condition, indicating the need for better understanding and management of non-urgent visits. A retrospective analysis study [36] explored the frequencies and characteristics of ED visits at King Abdulaziz University Hospital in Jeddah. The study found that the majority of ED visits were made by adults, with an admission rate of 23.44% and an overall ED mortality rate of 0.52%. Khalifa, 2015 [37] showed that only 11.2% of patients were admitted to the hospital inpatient departments, while the majority (85%) were discharged home after treatment. The study identified three main variables significantly influencing admission rates: patient acuity level, mode of arrival, and age group. Future analysis should focus on healthcare providers' compliance and adherence to the algorithm and the outcomes of treated, diverted, and referred patients. A descriptive exploratory study investigated the reasons for non-urgent presentations to the ED in Saudi Arabia. The survey revealed that many patients used the ED as their primary healthcare facility due to factors such as the lack of a primary care physician, the need for same-day treatment, and the availability of medical attention around the clock [38]. The study emphasized that several variables, including community understanding of the ED's function and access to primary healthcare providers, influence non-urgent visits.

Discussion

Our review points out that the demand for emergency care services is strong in Saudi Arabia, and this has led to overcrowding in emergency rooms, which might compromise patient treatment. Time spent waiting for care in the ED may be considerable, and patients who need to be admitted to the hospital typically have to wait much longer. Both the ED's flow and the overcrowding issue have been the subject of several efforts at resolution. Few solutions have been presented despite numerous efforts to do so. This report examines the factors that lead to overcrowding in Saudi Arabia's emergency rooms and discusses how those issues could be resolved. Overcrowding in emergency rooms may be caused by a number of factors, one of which is the shortage of physicians and nurses. The ED personnel is under pressure due to the high patient volume and the limited number of available staff members. Medical procedures, including X-rays, lab tests, and blood draws, take a lot of time. As a result, this causes congestion. Due to rising rates of accidents, illnesses including heart attack and neurological disorders, patient ignorance, and inadequate access to basic care, an increasing number of people are being brought to the ED.

In Saudi Arabia, emergency crowding is an important topic of research. It is a known reason associated with increasing the rate of patients being unseen, delaying treatment for patients who need urgent Care, and expanding the burnout of medical staff [39]. According to research [1] zone at a university hospital in Saudi Arabia, there is a correlation between the degree of ED congestion and the average duration of a patient's stay in the emergency room. A study of 350 people in KSA revealed that 63% did not have a regular healthcare provider and 62% wanted to obtain care on the same day and came because of the availability of medical care around the clock [38]. Over two-thirds of patients disagreed with the triage nurse's assessment of the severity of their illness [38]. The researchers came to the conclusion that most of these ED trips were caused by a lack of knowledge about the ED's function and an incorrect belief that primary care was unavailable [38]. A cross-sectional study was conducted at King Abdulaziz Hospital ER departments, King Fahd Hospital, and Althagor Hospital in November 2013 on 300 patients with an interview-based questionnaire and found that a higher proportion of patients visited the ED three to four times a year with non-urgent visits. Moreover, another cross-sectional study conducted between June 2016 and December 2018 shows that, out of 127,888 visits, 81% were non-urgent, and some of the patients requested ER services due to the limited services and working hours of PHC centers [17,40]. Through the articles that are included in our research, there are common reasons for emergency crowding that can be solved, such as shortage of medical staff, nurses, and doctors, shortage of equipment [39], lack of ED process, lack of patient knowledge about telemedicine services, and PHC [34], which lead to unsatisfactory services and can be associated with higher mortality rates [39]. A large number of patients would come to the ED for non-urgent causes. A cross-sectional study was done on 400 patients who visited the ED during morning shifts and showed that almost 78.5% of patients who visited the ED were non-urgent visits. Less urgent patients might be sent to primary care facilities, and the problem could be remedied with an urgency transfer strategy [33].

In Jeddah, a study was conducted to increase the number of intakes and decrease crowding. In the study, they stated some solutions to improve ED intake, first by using physician cubicles, and patients can be discharged from these cubicles or wait for results instead of occupying beds [41]. Additionally, you can decrease the time of taking personal information and past medical history by having electronic health records for the patients' status [41]. Process improvement by installing a system in EDs in Saudi Arabia to speed up the documentation [41]. The most common issue in emergency crowding is non-urgent visits. Therefore, telemedicine services such as medical call centers or smartphone applications have been proven to lessen the number of patients who intend to visit the ED [41]. A study that was conducted in 2020 shows that almost 50% of patients changed their intention to visit the ED after using telemedicine services [26,42]. The use of radio communication devices between the ED teams, doctors, and ambulances can help organize the patients' flow and the ambulance divergence and make it easier to contact the physician in urgent need of intervention [41]. In descriptive-analytical research at King Saud Hospital in AlQassim, they stated that the most affecting facets of ED crowding were health awareness and the number of beds in the facility [43]. Due to the importance of the topic and the lack of sufficient papers discussing the causes and solutions of ED overcrowding in Saudi Arabia, we conducted this systematic review to review all the available literature.

The number of beds in the ED is insufficient for many patients. The insufficiency is caused by the increased number of patients requiring emergency treatment with the slow flow. Patients have to wait for long hours to get free beds. Crowding in the ED causes an increased mortality rate. The highest mortality rate occurred during the overcrowding period for patients between 30 and 40 years. Many patients in the ED have long waiting hours; therefore, patients die waiting for medical services. The slowness of patient flow when getting checked in the ED makes the patients leave without receiving medical attention. It also affects the quality of care and the risk of adverse patient outcomes. The lack of awareness of ED rules among patients and not using telemedicine services affect the flow and quality of the health care service by increasing the number of non-urgent visits.

There are solutions for the crowding and flow problem of ED in Saudi Arabia. These are technology improvement, physical improvement, and process improvement. Advanced technology should be used to update the patient's health records. Past medical records of a patient should be moved automatically to the hospital a patient is in. Using telemedicine services decreases the number of patients who intend to visit the ED, resulting in reduced time consumption. In the process of improvement, triage should be used to determine the step of prioritization in the ED. The public should be aware of this process. On physical improvement, physician cubicles should be used to allow patients to be seen early by the doctor. There should be internal waiting areas for less acute patients, where they can sit waiting for results and treatment instead of occupying the insufficient beds. In addition, increasing the number of primary care centers and providing services for 24 hours will help improve health quality services and speed up the flow of ED. Since the leading identified causes relate to the number of people presenting to the ED, their discharge and bottlenecks in the ED system with high bed occupancy possibly play a role [6,7,44]. Solutions to ED included the following: A) adding a co-locating primary care center to reduce crowding depending on the role of primary care level visits to the ED, having a walk to the general practitioner center, or extending their hours; and B) adding a fast track for patients with less severe symptoms resulting in fewer wait times and increasing patient satisfaction [6,45,46]. Increasing ED beds and staff allows for lower wait times and faster discharge [6,7,46]. Strengths: This systematic review demonstrated a robust approach to exploring the causes, effects, and solutions of ED crowding in Saudi Arabia. We have strength points as the comprehensive search strategy and adherence to PRISMA guidelines ensured a thorough collection of relevant articles from reputable databases, providing a comprehensive overview of the topic. The inclusion criteria were clearly defined, focusing on recent and English-language studies, and the data extraction process involved multiple reviewers, ensuring the accuracy and consistency of information. Despite the strengths, this review may suffer from language bias, as it only considered English-language articles, potentially excluding relevant non-English publications. Additionally, the restriction to studies published in the last 10 years might have overlooked valuable insights from older research. The exclusion of grey literature and the potential for publication bias might limit the comprehensiveness of the findings. Lastly, the review's scope focused solely on Saudi Arabia, limiting its generalizability to other healthcare systems and regions with different contexts.

## Conclusions

ED crowding is becoming a major problem in the delivery of holistic care services in Saudi Arabia. High demand for emergency treatment should not be a hindrance to quality treatment. Physical, technological, and strategic measures should be put in place to fight the crowding problem in the ED in Saudi Arabia, as it may cause adverse effects such as transmission of diseases and deaths of patients. This systematic review revealed multiple factors behind the issue of ED crowding, such as a shortage of medical and nursing staff, the large influx of patients, insufficient beds in the ED, and the lack of integration of telemedicine services. As a result, the quality of care is compromised, and the medical staff is unable to deliver holistic care services to the patients. Long working hours also affect the care providers and cause work-related burnout. The lack of adequate care for the patients has been reported to cause adverse patient outcomes. In such a scenario, it is pertinent that the hospitals in Saudi Arabia integrate telemedicine services to ensure the smooth delivery of quality care services. Telemedicine is the way forward to reduce the burden of overcrowding in emergency departments.
